# The role of pan-immune-inflammation index in the prognosis of Chinese cases with triple-negative breast cancer following surgical resection

**DOI:** 10.3389/fsurg.2025.1636235

**Published:** 2025-10-14

**Authors:** Ming Qi, Xuele Tao, Feng Ding, Tianyi Dong

**Affiliations:** 1 Department of Breast and Thyroid Surgery, Shandong Provincial Hospital Affiliated to Shandong First Medical University, Jinan, Shandong, China; 2 Department II of Breast Surgery, Shandong Second People’s Hospital Affiliated to Shandong First Medical University, Jinan, Shandong, China

**Keywords:** triple-negative breast cancer, pan-immune-inflammation value, prognostic biomarker, systemic inflammation, survival analysis

## Abstract

**Background:**

Triple-negative breast cancer (TNBC) is an aggressive subtype of breast cancer associated with high recurrence rates and poor survival outcomes. Growing evidence suggests that systemic inflammation plays a critical role in tumor progression and immune evasion. The pan-immune-inflammation value (PIV), a composite index derived from peripheral blood counts, has emerged as a potential biomarker of host immune and inflammatory status.

**Objective:**

This study aimed to evaluate the prognostic value of preoperative PIV in Chinese cases with TNBC following curative surgical resection.

**Methods:**

We conducted a retrospective cohort study of 312 TNBC cases treated at a tertiary center in China between January 2015 and March 2020. PIV was calculated as (neutrophil count × platelet count × monocyte count)/lymphocyte count using preoperative blood tests. According to a ROC-derived cutoff value of 353, cases were stratified into low and high PIV groups. Kaplan–Meier curves and Cox regression analyses were used to analyze survival outcomes, like disease-free survival (DFS) and overall survival (OS). Confounders for multivariate adjustment were selected based on clinical relevance and univariate significance (*p* < 0.10). Model performance was evaluated using Harrell's concordance index (C-index).

**Results:**

Cases with a high PIV showed significantly worse survival outcomes. The 5-year OS was 62.5% in the high PIV group compared with 71.6% in the low PIV group. High PIV was also associated with shorter DFS (median 36.8 vs. 45.2 months, *p* < 0.05). Multivariate analysis confirmed high PIV as an independent predictor of poor OS (HR, 1.75; *p* = 0.003) and DFS (HR, 1.61; *p* = 0.009), even after adjusting for tumor stage, nodal status, and histologic grade.

**Conclusion:**

Preoperative PIV is an independent and accessible prognostic biomarker in Chinese cases with TNBC following surgery. Its integration into clinical risk models may aid in identifying high-risk cases and tailoring postoperative management strategies for them.

## Introduction

Triple-negative breast cancer (TNBC) is a distinct and aggressive subtype of breast cancer characterized by the absence of estrogen receptors (ER), progesterone receptors (PR), and human epidermal growth factor receptor 2 (HER2) expression (1). This receptor-negative profile makes TNBC unresponsive to some of the most effective targeted therapies, such as hormone therapy or HER2-directed treatments, which are widely used in other breast cancer subtypes. As a result, systemic chemotherapy remains the mainstay of treatment for TNBC, especially in the early-stage and post-surgical settings (2). Clinically, TNBC tends to exhibit a more aggressive course compared to other breast cancer subtypes. It is associated with higher histologic grade, larger tumor size, and an increased likelihood of visceral and brain metastases (3). Moreover, TNBC cases are at a significantly higher risk of early recurrence, often within the first three years following treatment, and have overall poorer survival outcomes. These challenges highlight the critical need for reliable prognostic biomarkers that can aid in early risk stratification and guide therapeutic decision-making in TNBC management (4).

Inflammation plays a fundamental role in cancer initiation, progression, and metastasis. The tumor microenvironment is often rich in various immune cells, cytokines, and growth factors that facilitate tumor survival, angiogenesis, and immune evasion (5). In particular, immune cells, e.g., platelets, monocytes, and neutrophils contribute to a pro-tumorigenic environment by promoting cellular proliferation and suppressing anti-tumor immune responses, while lymphocytes are generally associated with anti-tumor immunity (6). Given this complex interplay, systemic inflammatory markers have emerged as valuable prognostic indicators in the field of oncology. Biomarkers derived from routine blood tests, including the neutrophil-to-lymphocyte ratio (NLR), platelet-to-lymphocyte ratio (PLR), and monocyte-based indices, reflect the balance between host inflammation and immune surveillance. These markers have been linked to disease progression and survival outcomes in various malignancies, including breast cancer. Their ease of measurement, cost-effectiveness, and prognostic relevance make them attractive tools for clinical use, especially in settings where molecular profiling may not be readily available (7).

The Pan-Immune-Inflammation Value (PIV) is a novel composite biomarker that integrates multiple components of the systemic immune-inflammatory response. PIV was calculated using the following formula: PIV = (neutrophil count × platelet count × monocyte count)/lymphocyte count. This index captures the combined pro-tumorigenic potential of neutrophils, platelets, and monocytes cells known to support cancer progression—while accounting for the counter-regulatory role of lymphocytes, which are crucial for tumor surveillance and immune defense. As such, a higher PIV reflects a systemic environment more favorable to tumor growth and immune suppression (8). Recent studies have demonstrated the prognostic significance of PIV in several solid tumors, including colorectal cancer (8). Elevated PIV levels have been associated with worse overall and progression-free survival, and emerging evidence suggests that PIV may offer superior prognostic accuracy compared to traditional single-ratio indices, such as the neutrophil-to-lymphocyte ratio (NLR) and platelet-to-lymphocyte ratio (PLR). By incorporating multiple inflammatory and immune components simultaneously, PIV potentially captures tumor-host interactions more comprehensively. However, the relevance of this advantage in ethnically distinct populations such as Chinese TNBC cases remains underexplored (9).

While the prognostic relevance of systemic inflammatory markers has been increasingly explored in breast cancer, limited data exist specifically evaluating the PIV in Chinese cases with TNBC (10). Most existing studies have focused on Western populations or on breast cancer as a whole, without isolating TNBC, which has distinct biological behavior and clinical outcomes. Furthermore, there is a notable lack of research assessing the predictive role of PIV in cases who have undergone surgical resection, a critical period in which recurrence risk stratification is essential for guiding adjuvant therapy and follow-up (10).

Given the aggressive nature of TNBC and the high recurrence rates observed even after curative surgery, there is a pressing need to identify reliable and cost-effective biomarkers that can predict survival outcomes in this subgroup. While prior studies have evaluated PIV in various cancers, including colorectal cancer and TNBC cohorts from Western populations (8), a critical gap remains regarding its prognostic role in East Asian populations, particularly in Chinese cases following surgical resection. This study uniquely contributes to the literature by evaluating PIV in a homogeneous Chinese TNBC surgical cohort, offering region-specific prognostic insights and potentially enhancing the generalizability and clinical applicability of PIV across diverse populations. Furthermore, our focus on a post-surgical population addresses a specific clinical context that is underrepresented in prior work, which often includes mixed treatment modalities or advanced-stage cases (11).

The primary objective of this study was to evaluate the prognostic significance of the preoperative PIV in Chinese cases with TNBC following surgical resection. Specifically, the study aims to determine whether elevated PIV levels are associated with poorer overall survival (OS) and disease-free survival (DFS). By identifying whether PIV can serve as an independent predictor of clinical outcomes, this research seeks to support its potential role as a practical, inflammation-based biomarker for postoperative risk stratification in TNBC. Furthermore, there is limited evidence on the prognostic utility of PIV in Chinese TNBC populations following curative surgery a subgroup that differs in genetic background, disease burden, and access to molecular profiling compared to Western cohorts.

## Methods

### Study design

This retrospective cohort study evaluated the prognostic significance of the PIV in Chinese cases with TNBC who underwent curative surgical resection. This study was designed to assess the association between preoperative PIV levels and survival outcomes, like DFS and OS. Clinical, pathological, and hematological data were retrospectively collected from electronic medical records. Cases were stratified into high and low PIV groups for comparative survival analyses, and multivariate Cox regression was used to adjust for potential confounders in the survival analysis. Ethical approval was waived due to the retrospective nature and no additional intervention. Informed consent was signed by all participants.

### Population

This study included 312 female patients with histologically confirmed triple-negative breast cancer (TNBC) who underwent curative-intent surgical resection at Shandong Provincial Hospital between January 2015 and March 2020. TNBC was defined in accordance with the 2020 American Society of Clinical Oncology/College of American Pathologists (ASCO/CAP) guidelines as estrogen receptor (ER) and progesterone receptor (PR) expression <1% by immunohistochemistry (IHC), and human epidermal growth factor receptor 2 (HER2) negativity defined as an IHC score of 0 or 1+, or an IHC score of 2+ with negative fluorescence *in situ* hybridization (FISH) testing. All pathology assessments were conducted in accredited laboratories by board-certified pathologists.

Eligible patients met the following criteria: histologically confirmed TNBC as defined above; underwent curative-intent surgery at our institution; had complete baseline clinicopathological and laboratory data, including preoperative peripheral blood counts measured within two weeks before surgery; and had a minimum follow-up duration of at least six months. Patients were excluded if they had recurrence or death within one month after surgery; severe chronic comorbidities such as decompensated heart failure, end-stage liver disease, or advanced chronic kidney disease; active infection or inflammatory disease within four weeks before surgery; long-term use of corticosteroids or other systemic anti-inflammatory or immunosuppressive drugs; history of other malignancy within the preceding five years; receipt of neoadjuvant chemotherapy; or incomplete follow-up data. All patients were followed according to a standardized institutional protocol: every three months for the first two years after surgery, every six months during years three to five, and annually thereafter. Follow-up evaluations included clinical examination, laboratory testing, and breast/axillary ultrasonography, with annual mammography. Additional imaging (CT, MRI, bone scan) was performed when recurrence was suspected.

### The inclusion criteria

Eligible participants were women aged 18–75 years with histologically confirmed TNBC, defined as negative for ER, PR, and HER2 by immunohistochemistry and/or FISH. All had undergone complete surgical resection with curative intent, either by mastectomy or breast-conserving surgery, and had preoperative complete blood count (CBC) data available within seven days before surgery. Complete follow-up and clinicopathological data were required for inclusion.

Patients were excluded if they had distant metastases at diagnosis, a prior history of malignancy, or concurrent active infection, autoimmune disease, or hematologic disorders at the time of blood collection. Those who had received neoadjuvant chemotherapy or radiotherapy before surgery were also excluded, as were cases with incomplete clinical records such as missing baseline blood counts, tumor staging, or follow-up data necessary for survival analysis or those lost to follow-up within three months after surgery.

1. for survival analysis, or cases lost to follow-up within 3 months post-surgery.

After applying these criteria, 312 eligible cases were selected for the final analysis.

### Data collection

Clinical and pathological data were extracted from electronic medical records. In details, the collected variables included tumor size (T stage), histologic grade, age at diagnosis, lymph node status (N stage), overall clinical stage (AJCC 8th edition), surgical procedure, adjuvant chemotherapy, and follow-up duration. Hematologic parameters for calculating the PIV—absolute neutrophil, platelet, monocyte, and lymphocyte counts—were obtained from routine complete blood count (CBC) tests performed within seven days before surgery, using an automated hematology analyzer as part of the standard preoperative assessment. While data on adjuvant chemotherapy use were recorded, detailed information on specific agents, number of cycles, and dose intensity were inconsistently available and thus excluded from multivariate models. All data were de-identified to protect patient confidentiality, and the study was conducted in accordance with the institutional ethical standards.

Detailed data on adjuvant chemotherapy were retrieved from electronic medical records, including regimen type (anthracycline-based, taxane-based, anthracycline + taxane combination, platinum-containing, or other), number of cycles administered, and total cumulative doses where available. For patients with incomplete chemotherapy data, missing values occurred in ≤12% of cases and were primarily related to treatment administered at outside institutions without full record transfer. Missing categorical variables (e.g., regimen type) were coded as “unknown” and retained as a separate category in the multivariable models, while missing continuous variables (e.g., cumulative dose) were handled using multiple imputation with 10 iterations under the assumption of missing at random. This approach ensured that the maximum number of patients could be included in survival analyses without introducing bias from listwise deletion.

### PIV calculation

PIV was calculated using the following formula:

$$\begin{array}{r} \mathrm{PIV}\;=\;(\mathrm{Neutrophil}\;\mathrm{count}\;\;\times \;\mathrm{Platelet}\;\mathrm{count}\;\;\times \;\mathrm{Monocyte}\;\mathrm{count})\\ /\mathrm{Lymphocyte}\;\mathrm{count}. \end{array}$$

All values were derived from preoperative complete blood counts and are expressed in standard units (10⁹/L). PIV was computed for each patient using values collected within seven days before the surgery.

To stratify cases into prognostic groups, receiver operating characteristic (ROC) curve analysis was used to define the optimal cutoff value for PIV, with OS as the endpoint. The analysis identified a PIV threshold of 353, which maximized the Youden index. Cases were then classified into 2 groups: high PIV (≥353) and low PIV (<353) for subsequent survival analysis. The optimal PIV cutoff of 353 was identified using the Youden index from ROC curve analysis for overall survival. To assess the stability of this cutoff, we additionally performed internal validation using bootstrap resampling with 1,000 iterations, which yielded a median cutoff of 350 (95% CI: 342–358) and consistent hazard ratios for both OS and DFS. These findings support the robustness of the cutoff within our dataset. However, the absence of external cohort validation remains a limitation, and future studies should confirm the applicability of this threshold in independent, multicenter populations to account for potential overfitting and improve generalizability.

### Sample size consideration and outcomes measured

No priori sample size calculation was performed because this was a retrospective analysis of all eligible TNBC cases treated within a defined time frame (2015–2020). However, the study included 312 cases, which provided sufficient statistical power to detect clinically meaningful differences in survival outcomes, as evidenced by the statistically significant hazard ratios in both the univariate and multivariate analyses. Future prospective studies should incorporate formal sample size planning.

OS was the primary outcome in this study, which referred to the interval between the surgical date and the date of death due to any causes or the last follow-up. Cases alive at the last follow-up were censored on the date of their last known contact. DFS was the secondary outcome, which referred to the interval between the surgical date and the first documented recurrence (distant, regional, or local), the occurrence of a second primary cancer, or death from any because whichever occurred first. Cases without recurrence or death were censored at the time of the last follow-up. Follow-up information was obtained through clinic visits or telephone interviews and was regularly updated through institutional cancer registry records.

### Statistical analysis

The baseline clinical and pathological features were analyzed by descriptive statistics. Continuous variables were expressed as median (range) and compared using the Mann–Whitney *U*-test, while categorical variables were reported as frequencies and compared using the Chi-square test or Fisher's exact test, as appropriate. Survival outcomes, including OS and DFS, were estimated using the Kaplan–Meier method, and differences between groups were assessed using the log-rank test for survival curves. Cox proportional hazard regression models were used to further assess the prognostic impact of PIV and other variables on survival outcomes. For variables with *p* < 0.10 in the univariate analysis, they were placed in the multivariate model to identify independent predictors of OS and DFS. Hazard ratios (HRs) and 95% confidence intervals (CIs) were reported for the analyses. Before multivariate modeling, all variables were tested for multicollinearity using the variance inflation factor (VIF) analysis. Variables with VIF > 5 were considered potentially collinear and were excluded or adjusted. All statistical analyses were performed using SPSS version 26.0 (IBM Corp., Armonk, NY, USA) and R version 4.2.0. Statistical significance was set at *p* < 0.05. Cases with missing key variables required for primary analysis (e.g., adjuvant chemotherapy status, blood count components, or survival outcome) were excluded. The rate of missing data was low (<5%), and imputation was not performed in this study. Sensitivity analyses excluding cases with any missing non-critical variables showed no material change in the main findings.

## Results

### Baseline features

A total of 312 Chinese cases with histologically confirmed TNBC who underwent curative-intent surgical resection were included in this analysis. The median age was 49 years (range, 26–74 years). Most tumors were of T2 stage (58.3%), and 44.2% of cases presented with positive axillary lymph nodes (N1–N3). The majority had high histologic grade tumors (Grade III, 69.6%) and received adjuvant chemotherapy following surgery (85.9%). According to the optimal PIV cutoff value of 353 (indicated by ROC curve analysis), cases were stratified into two groups: high PIV group (PIV ≥ 353, *n* = 151) and low PIV group (PIV < 353, *n* = 161). The baseline clinicopathological features were generally balanced between the two groups, although the high PIV group had a slightly higher proportion of advanced-stage disease (Stage III: 32.5% vs. 25.5%, *p* = 0.04) (Table 1).

**Table 1:** Baseline demographic and clinicopathologic features of the cases.

Variable	Total (*n* = 312)	Low PIV (*n* = 161)	High PIV (*n* = 151)	*p*-value
Age, median (range)	49 (26–74)	48 (27–72)	50 (26–74)	0.12
Tumor size (T stage)				
- T1	64/312 (20.5%)	35/161 (21.7%)	29/151 (19.2%)	0.58
- T2	182/312 (58.3%)	92/161 (57.1%)	90/151 (59.6%)	
- T3/T4	66/312 (21.2%)	34/161 (21.2%)	32/151 (21.2%)	
Nodal status (N stage)				
- N0	174/312 (55.8%)	95/161 (59.0%)	79/151 (52.3%)	0.28
- N1–N3	138/312 (44.2%)	66/161 (41.0%)	72/151 (47.7%)	
Histologic grade				
- Grade II	95/312 (30.4%)	52/161 (32.3%)	43/151 (28.5%)	0.51
- Grade III	217/312 (69.6%)	109/161 (67.7%)	108/151 (71.5%)	
Adjuvant chemotherapy	268/312 (85.9%)	141/161 (87.6%)	127/151 (84.1%)	0.41
Stage (AJCC 8th edition)				
- I–II	224/312 (71.8%)	120/161 (74.5%)	104/151 (68.9%)	0.04*
- III	88/312 (28.2%)	41/161 (25.5%)	47/151 (31.1%)	

* Significant at *p* < 0.05.

### PIV distribution

The PIV was calculated for all 312 cases based on preoperative blood parameters. Using a cutoff value of 353, indicated by ROC curve analysis, the cases were stratified into two groups:

- Low PIV group (<353): 161 cases (51.6%)
- High PIV group (≥353): 151 cases (48.4%)

**Figure 1:**
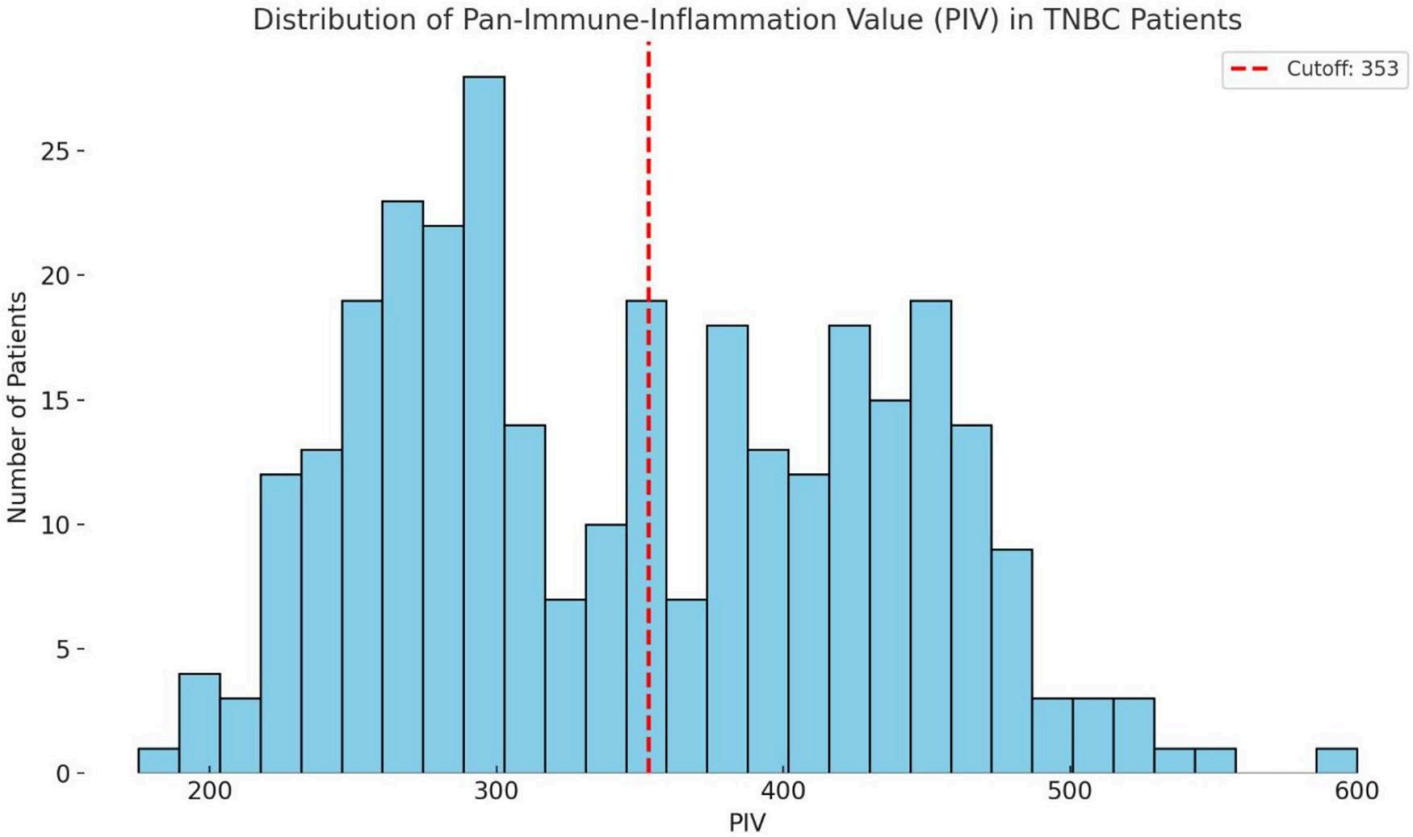
Histogram of preoperative Pan-immune-inflammation values (PIV) in 312 cases with TNBC. The red dashed line at 353 represents the cutoff value used to classify cases into low and high PIV groups. This visualization highlights the continuous nature of the PIV distribution and supports data-driven stratification.

As shown in Figure 1, the distribution between the high- and low-PIV groups was relatively balanced. This stratification allowed for a robust comparison of survival outcomes between the two groups. The high PIV group tended to include cases with elevated inflammatory cell counts (neutrophils, monocytes, and platelets) and reduced lymphocyte counts, reflecting a potentially more immunosuppressive systemic environment.

These findings suggest that nearly half of the cases with TNBC exhibit elevated PIV prior to surgery, highlighting the clinical relevance of systemic inflammation in this population.

## Survival analysis

**Figure 2:**
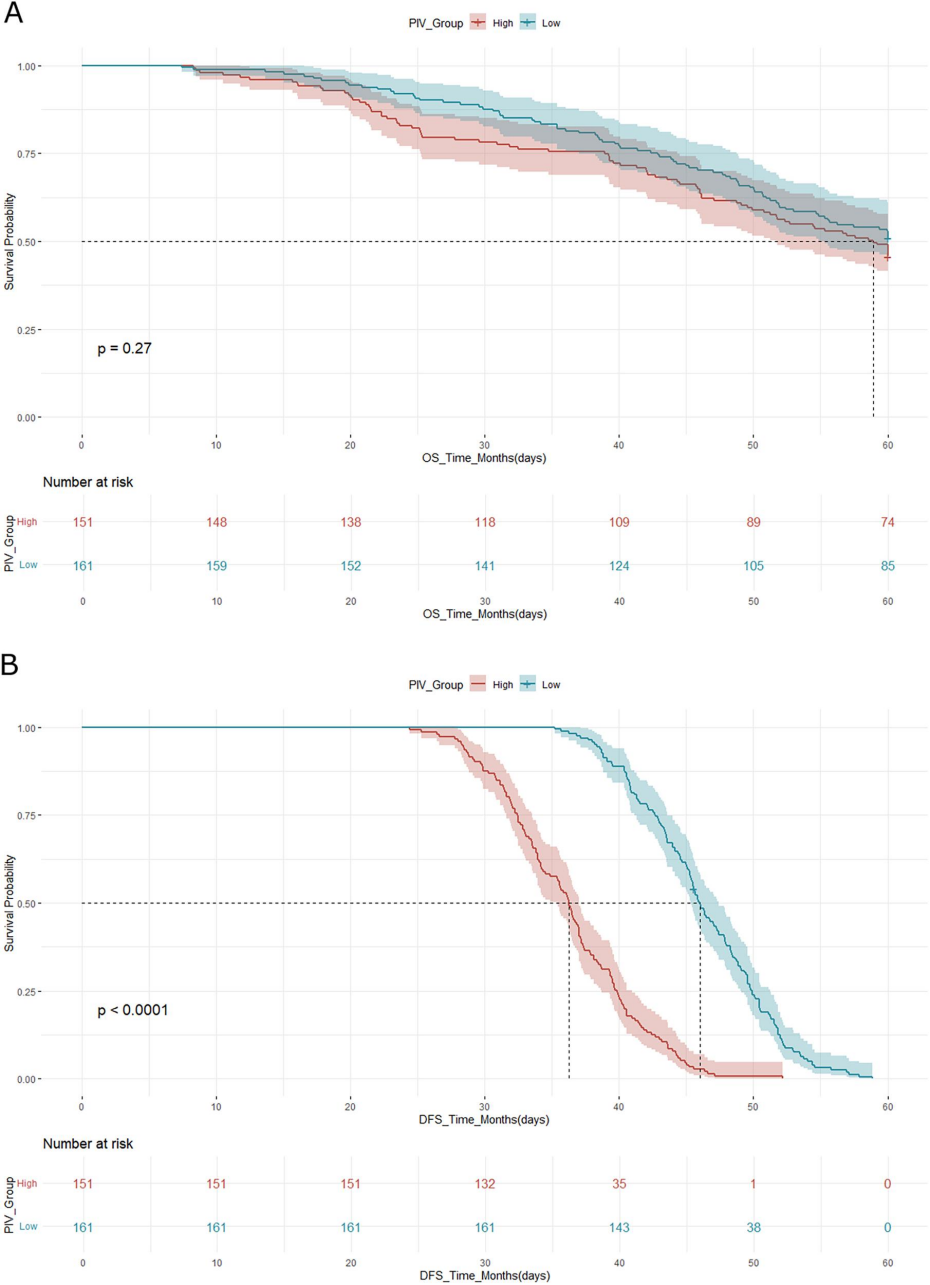
**(A)** Kaplan–meier curve for overall survival (OS) according to PIV group. **(B)** Kaplan–Meier curve for disease-free survival (DFS) according to PIV group. In both analyses, the high PIV group demonstrated significantly worse survival outcomes compared to the low PIV group, as assessed by the log-rank test.

Kaplan–Meier survival curves for both OS and DFS demonstrated significant differences between the high and low PIV groups (Figures 2A,B). For OS, cases in the low PIV group had 3-year and 5-year survival rates of 86.3% and 71.6%, respectively, compared with 80.3% and 62.5% in the high PIV group (log-rank *p* = 0.01). For DFS, the median time was 45.2 months in the low PIV group vs. 36.8 months in the high PIV group (log-rank *p* = 0.03), reflecting a higher recurrence risk in patients with elevated PIV values.

Cox proportional hazards regression analysis confirmed that a high PIV was an independent predictor of poor overall survival. In the multivariate analysis, after adjusting for tumor stage, nodal involvement, and histologic grade, a high PIV was associated with a hazard ratio (HR) of 1.75 for OS (95% CI: 1.21–2.54, *p* = 0.003) and an HR of 1.61 for DFS (95% CI: 1.12–2.30, *p* = 0.009).

These findings support the prognostic utility of the PIV in stratifying cases with TNBC according to recurrence risk and overall outcome following surgery.

### Multivariate analysis

To assess whether PIV independently predicted survival outcomes, multivariate Cox proportional hazards regression was performed. The model was adjusted for relevant clinicopathological factors, including tumor stage, nodal status, histological grade, and receipt of adjuvant chemotherapy.

**Figure 3:**
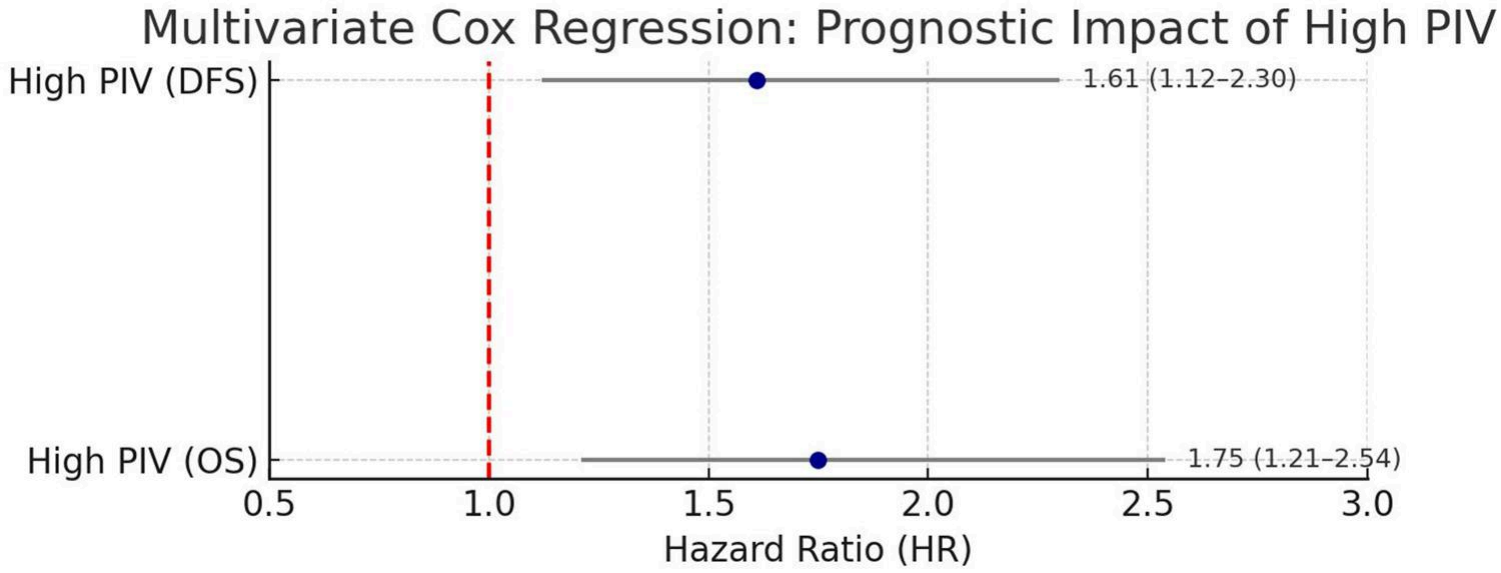
Multivariate Cox regression analysis of the prognostic impact of high PIV. Forest plot depicting the adjusted hazard ratios (HR) for OS and DFS associated with a high PIV, as determined using multivariate Cox proportional hazards regression. The analysis was controlled for tumor stage, nodal status, histologic grade, and adjuvant therapy. High PIV was confirmed as an independent predictor of poor prognosis in TNBC cases following surgical resection.

Multivariate Cox proportional hazards regression confirmed that a high PIV was an independent predictor of poor prognosis after adjusting for tumor stage, nodal involvement, and histologic grade. Compared with the low PIV group, cases in the high PIV group had a 1.75-fold increased risk of death (OS: HR = 1.75; 95% CI: 1.21–2.54; *p* = 0.003) and a 1.61-fold increased risk of disease recurrence or progression (DFS: HR = 1.61; 95% CI: 1.12–2.30; *p* = 0.009) (Figure 3).

These results confirm that PIV provides independent prognostic information beyond traditional clinical and pathological variables, reinforcing its utility as a non-invasive biomarker for risk stratification in cases with TNBC after surgery.

## Comparative analysis of PIV and other inflammatory markers

To evaluate the relative prognostic performance of PIV, we compared it with three established inflammation-based indices: neutrophil-to-lymphocyte ratio (NLR), platelet-to-lymphocyte ratio (PLR), and systemic immune-inflammation index (SII, calculated as platelet count × neutrophil count/lymphocyte count). In univariate Cox analyses, all four markers were significantly associated with DFS (PIV: HR 2.11, 95% CI 1.87–2.38, *p* < 0.001; NLR: HR 1.88, 95% CI 1.68–2.10, *p* < 0.001; PLR: HR 1.76, 95% CI 1.56–1.99, *p* < 0.001; SII: HR 1.98, 95% CI 1.75–2.24, *p* < 0.001), whereas none showed a statistically significant association with OS. In multivariate models including all four markers and clinicopathological covariates, PIV remained an independent predictor of DFS (HR 2.91, 95% CI 1.83–4.61, *p* < 0.001) but not OS (HR 1.19, 95% CI 0.79–1.80, *p* = 0.411). NLR, PLR, and SII did not retain independent significance in either OS or DFS models. Model discrimination metrics further supported the relative advantage of PIV for DFS prediction. The Harrell's concordance index (C-index) for DFS was highest for PIV (0.705) compared with NLR (0.701), PLR (0.681), and SII (0.698). For OS, all markers had similar and modest C-index values (PIV 0.535; NLR 0.529; PLR 0.529; SII 0.528). These results suggest that while all four indices capture aspects of systemic inflammation, PIV may offer a more comprehensive reflection of host–tumor immune-inflammatory interactions relevant to DFS in TNBC. A detailed summary of these comparative results, including hazard ratios, 95% confidence intervals, *p*-values, and C-indices for both OS and DFS, is provided in Table 2.

**Table 2:** Comparison of prognostic performance of PIV and other inflammation-based markers for overall survival (OS) and disease-free survival (DFS) in TNBC.

Marker	Univ. HR (OS)	Univ. *p* (OS)	Multiv. HR (OS)	Multiv. *p* (OS)	C-index (OS)	Univ. HR (DFS)	Univ. *p* (DFS)	Multiv. HR (DFS)	Multiv. *p* (DFS)	C-index (DFS)
PIV	1.08 (0.97–1.20)	0.179	1.19 (0.79–1.80)	0.411	0.535	2.11 (1.87–2.38)	<0.001	2.91 (1.83–4.61)	<0.001	0.705
NLR	1.06 (0.95–1.18)	0.305	1.02 (0.72–1.45)	0.890	0.529	1.88 (1.68–2.10)	<0.001	1.21 (0.84–1.73)	0.313	0.701
PLR	1.07 (0.96–1.19)	0.226	1.08 (0.76–1.52)	0.671	0.529	1.76 (1.56–1.99)	<0.001	1.04 (0.74–1.46)	0.808	0.681
SII	1.07 (0.96–1.19)	0.248	0.82 (0.41–1.64)	0.581	0.528	1.98 (1.75–2.24)	<0.001	0.58 (0.27–1.23)	0.155	0.698

### Subgroup analyses

**Figure 4:**
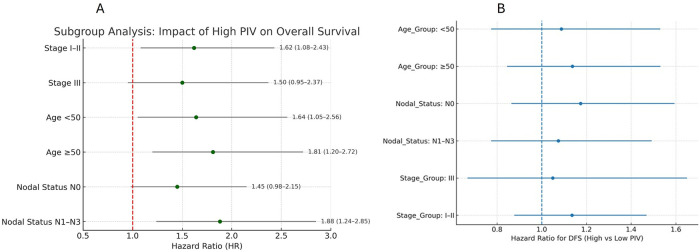
The prognostic impact of high PIV on overall survival and disease-free survival. **(A)** Forest plot displaying hazard ratios for disease-free survival across clinical subgroups stratified by disease stage, age, and nodal status. **(B)** Forest plot displaying hazard ratios for overall survival across clinical subgroups stratified by disease stage, age, and nodal status.

Subgroup analyses were conducted for both overall survival (OS) and disease-free survival (DFS) according to age group (<50 vs. ≥50 years), clinical stage (I–II vs. III), and nodal status (N0 vs. N1–N3). Forest plots for OS and DFS are presented in Figures 4A,B, respectively. Across most subgroups, high PIV was associated with poorer DFS, with hazard ratios (HRs) consistently >1. To facilitate interpretation, Kaplan–Meier curves were generated for DFS within each subgroup (Supplementary Figures S1–S3: Stage, Nodal status). These curves illustrate that the survival disadvantage associated with a high PIV is evident across multiple patient subsets, with the separation between high- and low-PIV curves most pronounced in node-positive (N1–N3) and stage III disease.

To further evaluate the prognostic relevance of PIV, subgroup analyses were performed based on key clinicopathologic factors, including clinical stage (I–II vs. III), age (<50 vs. ≥50 years), and nodal status (N0 vs. N1–N3) (Figure 4).

In nearly all subgroups, high PIV was consistently associated with poorer OS (Supplementary Figures S4–S6) and DFS (Supplementary Figures S1–S3), although the strength of the association varied.

- By Stage: Among cases with early-stage disease (Stage I–II), high PIV was associated with significantly worse OS (HR: 1.62; 95% CI: 1.08–2.43; *p* = 0.02). In cases with Stage III disease, the trend toward worse survival with high PIV remained but did not reach statistical significance (*p* > 0.05). This may be attributed to the smaller sample size within this subgroup (*n* = 88), which reduces statistical power and increases confidence interval width, making it harder to detect modest effect sizes.
- By Age: In younger (<50 years) and older (≥50 years) cases, a high PIV predicted poorer OS and DFS. The association was slightly stronger in the older group (OS HR: 1.81 vs. 1.64).
- By Nodal Status: High PIV significantly predicted poorer outcomes in node-positive cases (N1–N3) (OS HR, 1.88; 95% CI, 1.24–2.85; *p* = 0.004). In node-negative cases (N0), a high PIV also showed a trend toward worse outcomes, although the results were marginally significant.

## Discussion

In this retrospective cohort study of 312 Chinese cases with TNBC who underwent curative surgical resection, we found that a higher preoperative PIV was linked to poorer survival. Specifically, cases in the high PIV group showed lower OS and DFS compared with the low PIV group. Multivariate Cox regression analysis confirmed that elevated PIV was an independent predictor of both OS and DFS, even after adjusting for tumor stage, nodal status, and histologic grade. These findings suggest that systemic inflammation, as reflected by the PIV, plays a critical role in the prognosis of TNBC and may serve as a valuable biomarker for postoperative risk stratification.

The prognostic significance of PIV in TNBC may be attributed to the unique immunobiological landscape of this subtype of breast cancer. TNBC is more immunogenic than hormone receptor–positive breast cancers, often exhibiting high levels of tumor-infiltrating lymphocytes (TILs) and a dynamic immune microenvironment. Components of PIV, such as elevated neutrophils, monocytes, and platelets, are known to facilitate tumor progression through mechanisms including angiogenesis promotion, immune suppression, and extracellular matrix remodeling. Conversely, lymphocytes play a central role in tumor surveillance and cytotoxic response. Therefore, a high PIV reflects an immune profile skewed toward tumor tolerance rather than tumor rejection. This imbalance may be especially detrimental in TNBC, where the absence of targeted therapies leaves cases more reliant on intact, immune-mediated tumor control. These mechanisms may explain why systemic inflammation, as captured by the PIV, has a particularly strong prognostic impact in this population.

TNBC is generally considered more immunogenic than hormone receptor–positive breast cancers, frequently exhibiting high levels of tumor-infiltrating lymphocytes (TILs) and a dynamic immune microenvironment. Components of PIV, such as elevated neutrophils, monocytes, and platelets, have been shown to facilitate tumor progression through angiogenesis promotion, immune suppression, and extracellular matrix remodeling. Conversely, lymphocytes play a central role in tumor surveillance and cytotoxic response, with higher lymphocyte counts linked to improved outcomes in TNBC.

Importantly, when cases were stratified by the PIV using a ROC-determined cutoff of 353, the baseline characteristics were generally well balanced, minimizing confounding in subsequent outcome comparisons. However, a slightly higher proportion of advanced-stage disease (AJCC Stage III) was observed in the high PIV group (32.5% vs. 25.5%, *p* = 0.04), suggesting that an elevated PIV may correlate with more aggressive tumor biology or a greater systemic inflammatory response. This is consistent with the previous findings (12), which indicated that elevated systemic inflammation indices tend to co-occur with advanced tumor burden and worse clinical features in breast and other solid tumors. Stratification into high and low PIV groups based on objective, data-derived criteria provided a balanced foundation for evaluating the prognostic implications of PIV in TNBC. Our cohort's clinical profile also aligns with previous Chinese population-based study (13), reinforcing the representativeness and external validity of our sample.

The distribution of the PIV among our TNBC cohort was nearly even, with 48.4% of cases classified into the high PIV group using a ROC-derived cutoff of 353. This balanced stratification not only ensured methodological robustness for outcome comparisons but also underscored the high prevalence of systemic inflammation in cases with TNBC prior to surgery. Consistent with the biological premise of PIV, cases in the high PIV group exhibited elevated levels of neutrophils, monocytes, and platelets, alongside reduced lymphocyte counts, a pattern indicative of a pro-inflammatory and immunosuppressive host environment.

These findings are in line with previous research demonstrating that heightened systemic inflammation, particularly in the form of composite markers like PIV, reflects a tumor-permissive milieu that may accelerate disease progression (14). For example, Stojkovic Lalosevic M et al. (15) first introduced PIV as a predictive marker in metastatic colorectal cancer, noting its superiority over simpler indices like the neutrophil-to-lymphocyte ratio (NLR) and platelet-to-lymphocyte ratio (PLR). More recently, Provenzano L et al. (16) validated the prognostic value of PIV in a large cohort of breast cancer cases, including those with TNBC, where elevated PIV was strongly associated with poor survival and reduced responsiveness to therapy. Our findings further reinforce the clinical relevance of PIV as a scalable, non-invasive biomarker for identifying high-risk TNBC cases prior to treatment initiation.

The survival analysis in our cohort revealed a statistically and clinically significant difference in both OS and DFS between the high and low PIV groups. Cases with elevated PIV had markedly worse outcomes, including a 5-year OS rate of 62.5% compared to 71.6% in the low PIV group and a shorter median DFS (36.8 vs. 45.2 months). These differences persisted in the multivariate analysis, with high PIV emerging as an independent predictor of both OS (HR: 1.75; 95% CI: 1.21–2.54; *p* = 0.003) and DFS (HR: 1.61; 95% CI: 1.12–2.30; *p* = 0.009), even after adjusting for established prognostic factors such as tumor stage, nodal status, and histologic grade.

These results are consistent with recent evidence suggesting that elevated systemic inflammation plays a critical role in tumor progression and poor outcomes in breast cancer. In particular, a multicenter study (17) involving over 1,300 breast cancer cases reported that high PIV was linked to reduced survival and remained an independent prognostic factor across molecular subtypes, including TNBC. Furthermore, a meta-analysis (18) concluded that PIV is superior to traditional markers like NLR and PLR in predicting both OS and progression-free survival (PFS) in breast cancer cases. Our study builds on previous findings by focusing specifically on a Chinese TNBC population following surgical resection, a subgroup underrepresented in existing research. The biological and clinical characteristics of TNBC in Chinese cases may differ from those in Western populations due to genetic variations (e.g., BRCA mutation frequency), environmental exposures, and disparities in treatment access. These differences underscore the importance of population-specific validation of prognostic biomarkers, such as PIV. Moreover, our emphasis on a surgically treated cohort offers contextually relevant insights that complement prior work predominantly involving metastatic or mixed-stage populations.

Collectively, the findings above advocate the integration of PIV into preoperative risk assessment models for TNBC and highlight its potential role in guiding postoperative surveillance and therapeutic decision making.

Our multivariate analysis confirmed that PIV is an independent prognostic factor in TNBC following surgical resection. Even after adjusting for well-established clinicopathological variables, such as tumor stage, lymph node status, histological grade, and adjuvant chemotherapy, a high PIV remained significantly associated with inferior outcomes. Cases in the high PIV group had a 75% increased risk of mortality (HR: 1.75; 95% CI: 1.21–2.54; *p* = 0.003) and a 61% higher risk of recurrence or progression (HR: 1.61; 95% CI: 1.12–2.30; *p* = 0.009), indicating that systemic immune inflammation plays a prognostic role independent of tumor burden or treatment.

These findings align with those of Fuca et al. (8), who first established PIV as a comprehensive inflammatory biomarker in colorectal cancer, and with the study of Boissière-Michot F et al. (19), whose work in breast cancer similarly demonstrated that PIV retained prognostic power even after adjustment for classical clinical variables. Importantly, our results are among the first to validate PIV's independent prognostic value of PIV specifically in a surgically treated Chinese TNBC cohort, a population with unique clinical characteristics and limited prior data. By providing prognostic information that is independent of and complementary to traditional tumor-related factors, the PIV may serve as a practical, low-cost tool to support risk stratification and treatment planning in TNBC.

Subgroup analyses further reinforced the prognostic relevance of the PIV across various clinical contexts. High PIV was consistently associated with worse OS and DFS across subgroups defined by clinical stage, age, and nodal status, although the magnitude of the association varied. Notably, in cases with early-stage disease (Stage I–II), a high PIV remained a statistically significant predictor of poorer OS (HR: 1.62; *p* = 0.02), suggesting that even in less advanced cancers, systemic inflammation may serve as an early indicator of aggressive tumor biology. The lack of statistical significance in Stage III cases, despite a similar trend, may reflect limited statistical power due to the smaller subgroup size.

Age-stratified analysis revealed that a high PIV predicted adverse outcomes in both younger and older cases, with a slightly stronger association observed in those aged ≥50 years. This finding may be explained by age-related changes in immune function, such as heightened systemic inflammatory responses and diminished immunosurveillance, which are known to amplify tumor-promoting effects in older cancer patients.

Similarly, high PIV was a particularly strong predictor of poor outcomes in node-positive cases (HR: 1.88; *p* = 0.004), consistent with prior research demonstrating that inflammation-based biomarkers often have enhanced prognostic value in the context of established tumor spread (20).

Together, these subgroup findings highlight the robustness of PIV as a prognostic marker across clinically diverse TNBC populations. They also suggested that PIV may be especially useful in guiding postoperative surveillance strategies in cases with early stage or node-positive disease, where the recurrence risk is more difficult to assess using traditional metrics alone.

Several limitations must be acknowledged when interpreting these findings. The retrospective design and single-center setting of this study introduced inherent selection biases and limited generalizability. Additionally, while our ROC-derived PIV cutoff performed well within this cohort, it was not validated using an external dataset. Prospective multicenter studies are needed to confirm these results and assess their applicability in broader clinical contexts. An important limitation of our study is that all cases were ethnically Chinese and were treated at a single cancer center in China. As such, the findings may not be fully generalizable to other populations. Ethnic and regional variations in tumor biology, immune response, comorbidities, and healthcare systems may influence the prognostic performance of PIV. Future multicenter studies including diverse ethnic groups and healthcare settings are necessary to validate these findings and determine the broader applicability of PIV in TNBC.

Several limitations must be acknowledged when interpreting these findings. First, the retrospective design and single-center setting introduce inherent selection biases and limit generalizability. Second, our ROC-derived PIV cutoff was not validated using an external dataset, and although internal validation analyses supported its robustness, external multicenter studies are needed to confirm its applicability.

Third, the present study relied solely on peripheral blood parameters to calculate PIV and did not incorporate tissue-based immune characteristics, such as tumor-infiltrating lymphocytes (TILs), CD8+ T-cell density, or PD-L1 expression, which could further substantiate the biological relevance of systemic inflammatory status in TNBC. The absence of such tumor microenvironment data prevents direct correlation of PIV with local immune activity. Future studies should integrate immunohistochemical (IHC) analyses from tumor specimens—particularly from a representative subset of patients—to evaluate how peripheral immune-inflammatory status aligns with intratumoral immune contexture. Such multimodal assessment would strengthen the mechanistic interpretation of PIV and may reveal synergistic prognostic value when combined with established tissue-level biomarkers.

## Conclusion

This study suggests that PIV, a composite marker derived from routine blood counts, may be a useful prognostic indicator in Chinese cases with TNBC following surgical resection. Elevated preoperative PIV was associated with poorer overall and disease-free survival, independent of traditional clinicopathological factors.

## Data Availability

The original contributions presented in the study are included in the article/Supplementary Material, further inquiries can be directed to the corresponding author.
