# Supplementary material for: The role of pan-immune-inflammation index in the prognosis of Chinese cases with triple-negative breast cancer following surgical resection
Source: Front Surg. 2025 Oct 14;12:1636235. doi: 10.3389/fsurg.2025.1636235 (PMC12558987; doi:10.3389/fsurg.2025.1636235)
Supplement: Supplementary file 1 [file Supplementaryfile1.docx]

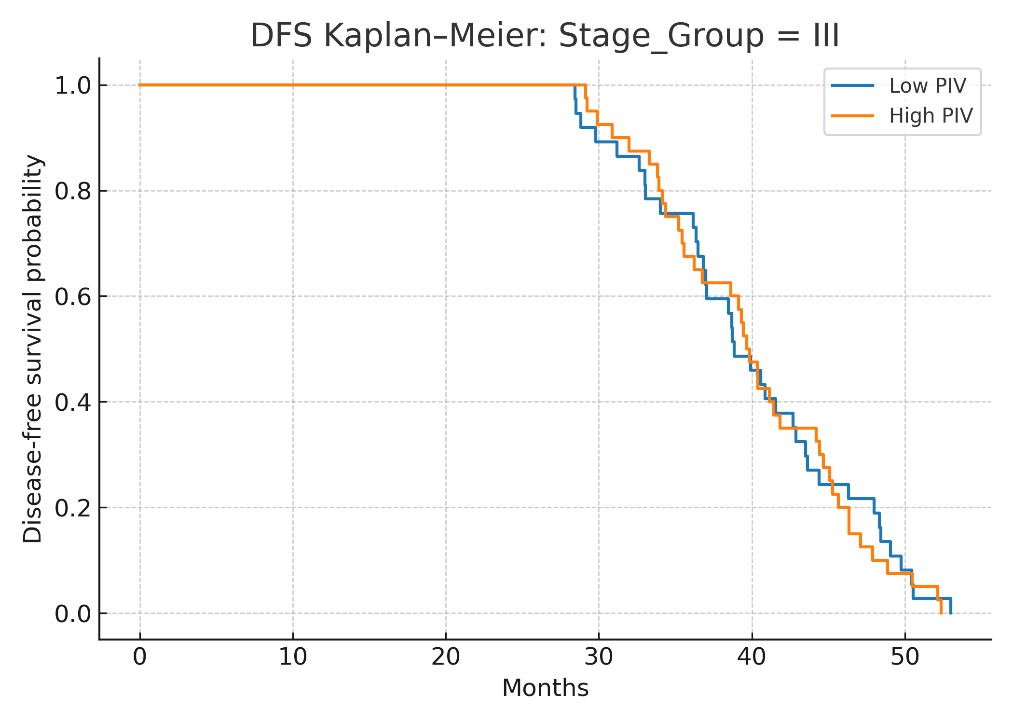


Figure S1


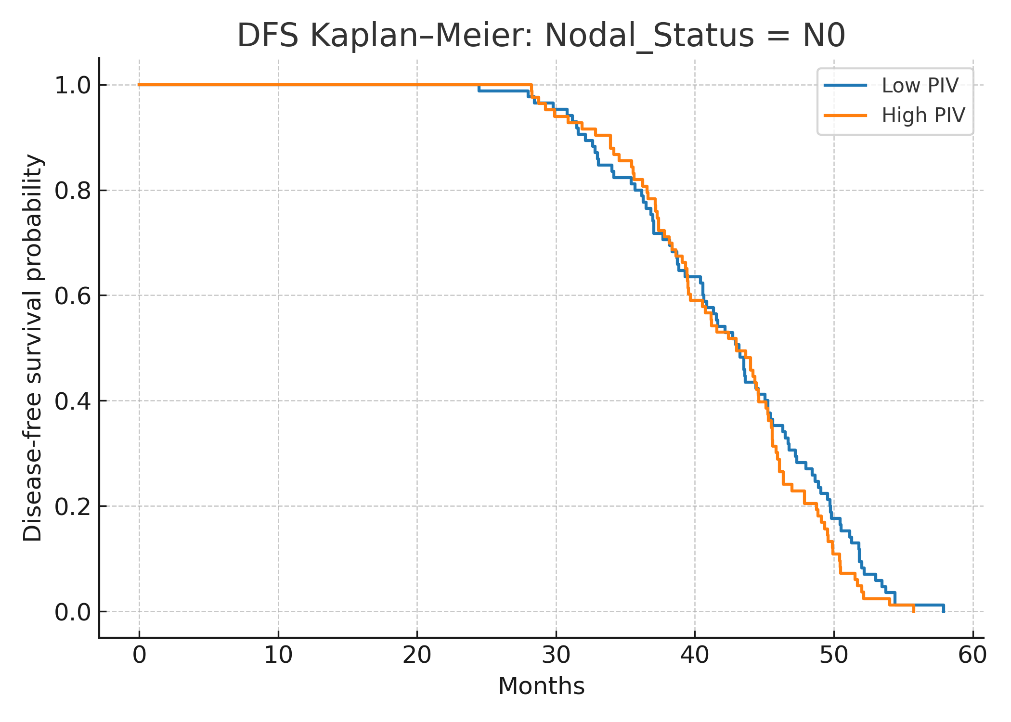


Figure S2


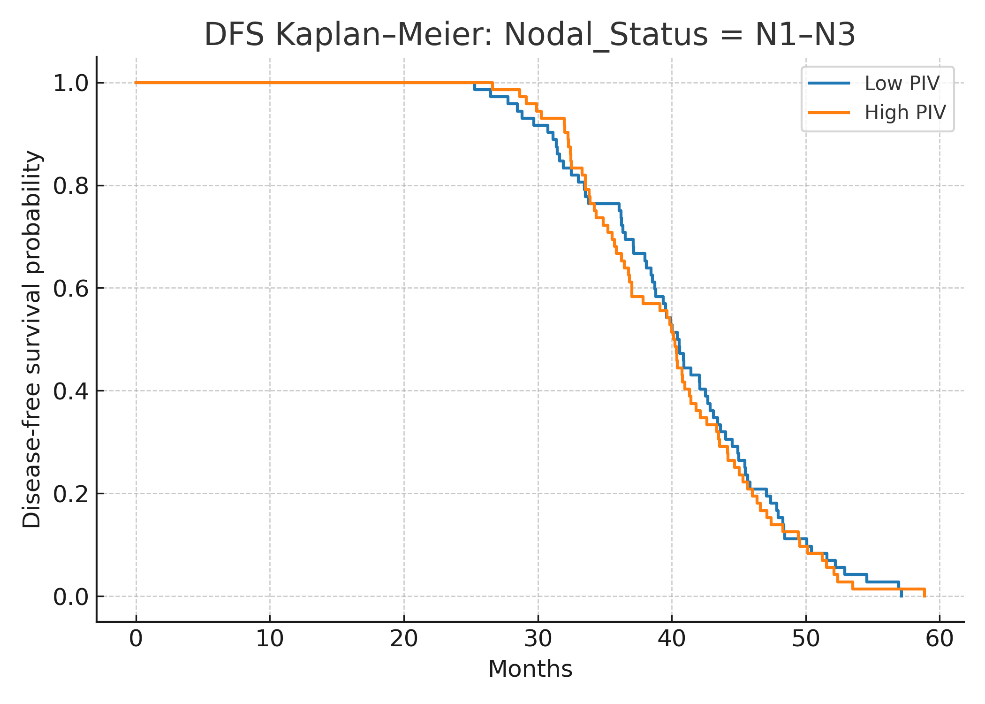


Figure S3

OS Kaplan–Meier: Stage_Group = III


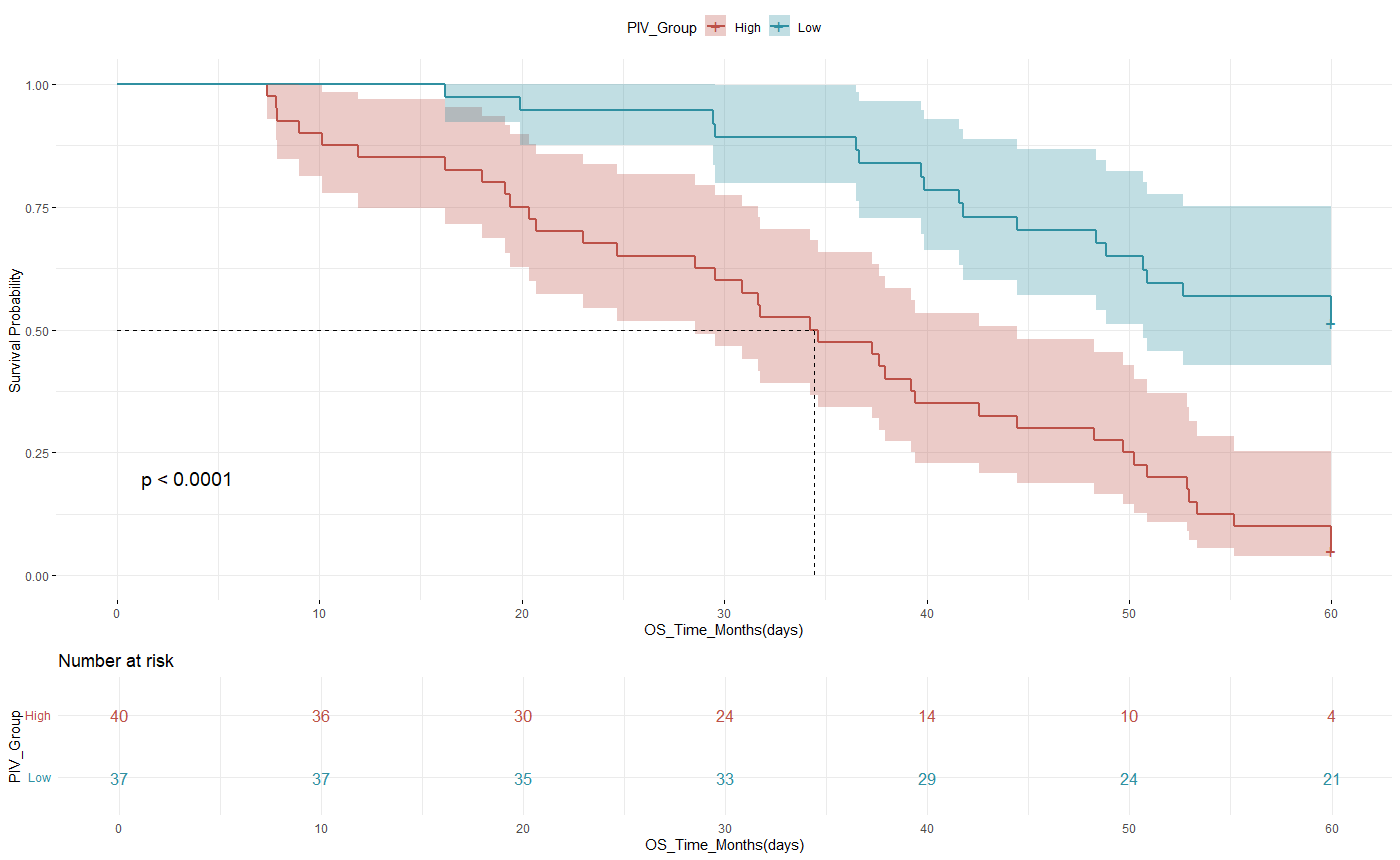


Figure S4

OS Kaplan–Meier: Nodal_Status = N0


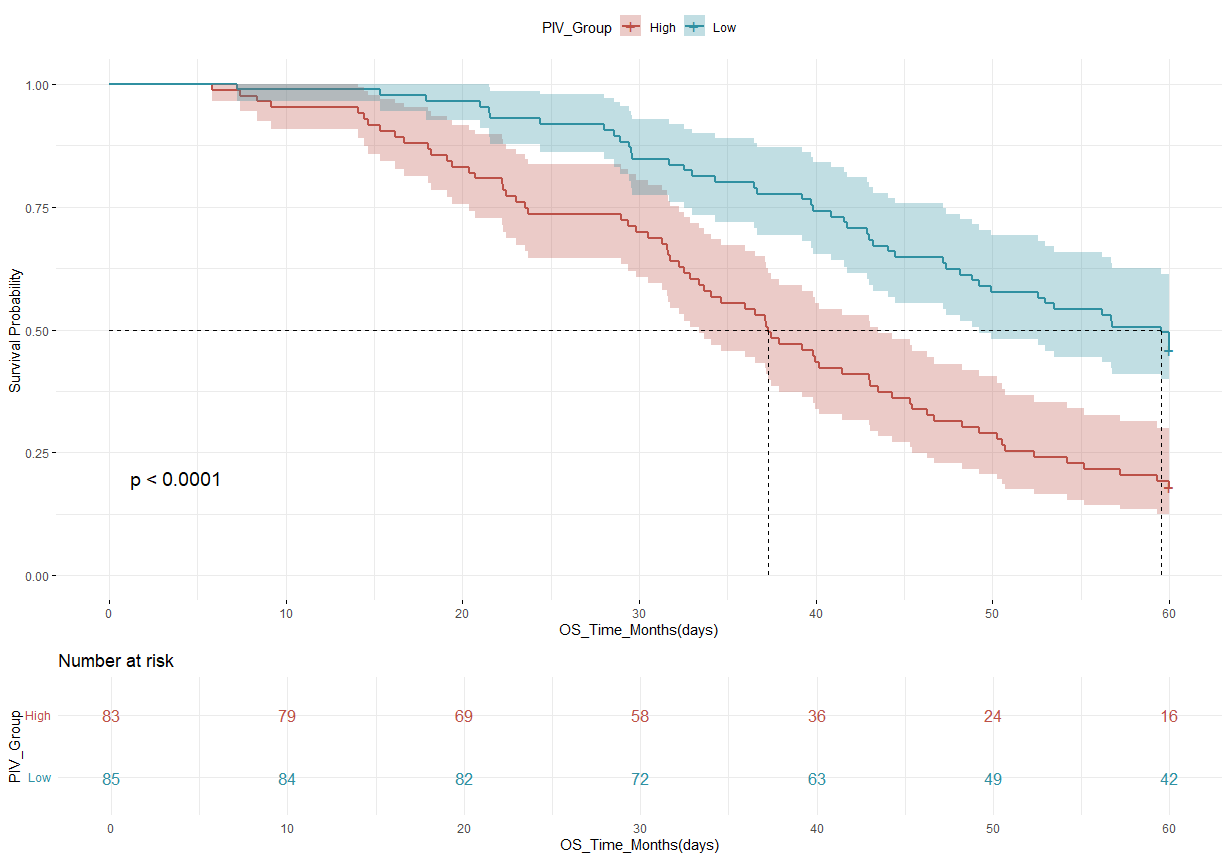


Figure S5

OS Kaplan–Meier: Nodal_Status = N1-N3


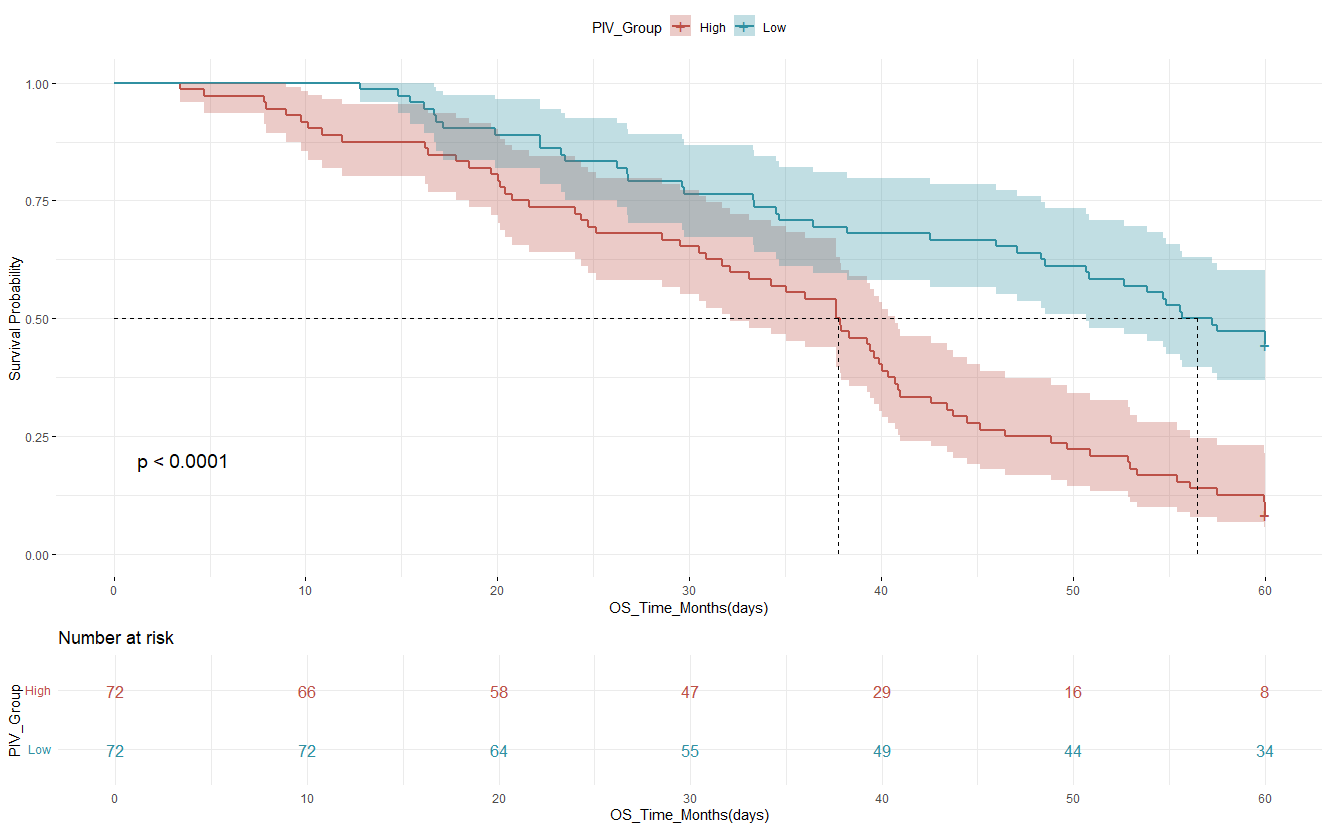


Figure S6
